# Parkin regulates kainate receptors by interacting with the GluK2 subunit

**DOI:** 10.1038/ncomms6182

**Published:** 2014-10-15

**Authors:** AnnaMaria Maraschi, Andrea Ciammola, Alessandra Folci, Francesca Sassone, Giuseppe Ronzitti, Graziella Cappelletti, Vincenzo Silani, Shigeto Sato, Nobutaka Hattori, Michele Mazzanti, Evelina Chieregatti, Christophe Mulle, Maria Passafaro, Jenny Sassone

**Affiliations:** 1 IRCCS Istituto Auxologico Italiano, Department of Neurology and Laboratory of Neuroscience, Cusano Milanino, 20095 Milan, Italy; 2 CNR Institute of Neuroscience, Department of BIOMETRA, University of Milan, 20129 Milan, Italy; 3 Department of Neuroscience and Brain Technologies, Istituto Italiano di Tecnologia, 16163 Genova, Italy; 4 Department of Biosciences, Università degli Studi di Milano, 20122 Milan, Italy; 5 ‘Dino Ferrari’ Center, Department of Pathophysiology and Transplantation, Università degli Studi di Milano, 20122 Milan, Italy; 6 Department of Neurology, Juntendo University School of Medicine, 113-8421 Tokyo, Japan; 7 Interdisciplinary Institute for Neuroscience, CNRS UMR 5297, University of Bordeaux, 33000 Bordeaux, France

## Abstract

Although loss-of-function mutations in the *PARK2* gene, the gene that encodes the protein parkin, cause autosomal recessive juvenile parkinsonism, the responsible molecular mechanisms remain unclear. Evidence suggests that a loss of parkin dysregulates excitatory synapses. Here we show that parkin interacts with the kainate receptor (KAR) GluK2 subunit and regulates KAR function. Loss of parkin function in primary cultured neurons causes GluK2 protein to accumulate in the plasma membrane, potentiates KAR currents and increases KAR-dependent excitotoxicity. Expression in the mouse brain of a parkin mutant causing autosomal recessive juvenile parkinsonism results in GluK2 protein accumulation and excitotoxicity. These findings show that parkin regulates KAR function *in vitro* and *in vivo*, and suggest that KAR upregulation may have a pathogenetic role in parkin-related autosomal recessive juvenile parkinsonism.

## Abstract (web summary)

Loss-of-function mutations in the *PARK2* gene are implicated in autosomal recessive juvenile parkinsonism, but the mechanisms are unclear. Here, the authors show that these mutations cause accumulation of the kainate receptor subunit GluK2 in the plasma membrane of neurons, which facilitates neuronal death.

## Graphical abstract

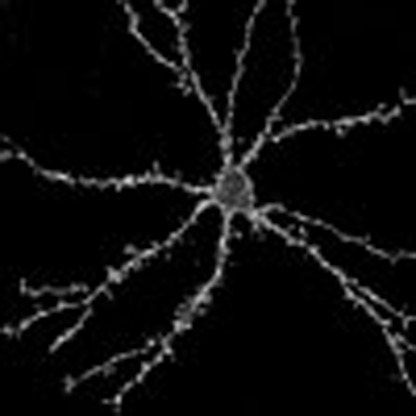

Although loss-of-function mutations in the *PARK2* gene cause the most common form of autosomal recessive juvenile parkinsonism (ARJP)^1^,^2^, the underlying molecular mechanism remains unclear. The *PARK2* gene encodes the parkin protein, an ubiquitin E3 ligase widely expressed throughout the central nervous system whose functions include targeting proteins for degradation^3^ and modulating mitochondria turnover^4^,^5^. Beside these well-recognized functions, increasing evidence suggests that parkin also regulates excitatory synapses. Immunofluorescence studies have detected widespread parkin expression in neurons, including dendrites and spines^6^. The carboxy-terminal domain of parkin contains a class II PDZ-binding motif that binds the protein CASK in postsynaptic densities^7^. Parkin associates with and ubiquitinates the PDZ protein PICK1, thus modulating the activity of ion channels involved in synaptic plasticity^8^. Parkin also mediates endophilin-A1 ubiquitination and some evidence suggests that endophilin-A-mediated parkin recruitment promotes synaptic protein ubiquitination^9^. The loss of endogenous parkin sensitizes *in vitro* midbrain dopaminergic neurons to kainate toxicity^10^, increases excitatory glutamatergic synapse numbers and increases vulnerability to synaptic excitotoxicity in cultured hippocampal neurons^6^. Hence, although current knowledge strongly suggests that parkin intervenes in modulating glutamatergic synapse function, the underlying molecular mechanism is unknown. We hypothesized that parkin may directly regulate ionotropic glutamate receptors of the *N*-methyl-D-aspartate (NMDAR), α-amino-3-hydroxy-5-methyl-4-isoxazolepropionic acid (AMPAR) or kainate receptor (KAR) types. Our results show that parkin regulates KAR function and suggest that KAR may have a pathogenetic role in parkin-related ARJP.

## Results

### Increased GluK2 levels in *PARK2* brain tissues

**Figure 1:**
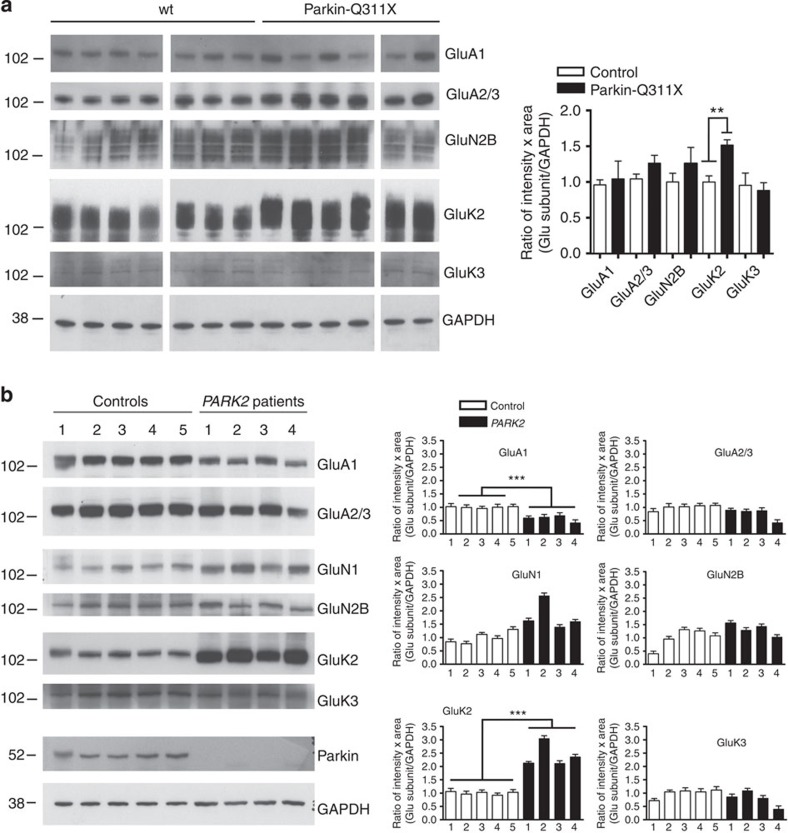
Increased GluK2 levels in brain tissues from the *PARK2* mouse model parkin-Q311X and from patients with *PARK2* mutations. (**a**) Western blottings of glutamate receptor subunits in substantia nigra lysates from controls (*n*=7) and littermate parkin-Q311X mice (*n*=6). All mice were 3 to 4 weeks old. Samples were run in parallel gels, each gel contained wt and parkin-Q311X samples. Western blottings were blotted and developed contemporaneously. Results derive from three independent experiments. The densitometer analysis shows that GluK2 protein is significantly increased in parkin-Q311X mice as compared with littermate controls (unpaired *t*-test, **P=0.0010, *t*=4.424). No statistically significant differences were found for the other AMPA, NMDA and KAR subunits analysed (unpaired *t*-test, *P*>0.05). No GluK1 and GluN1 expression was detectable. Error bars indicate±s.e.m. (**b**) Western blottings for glutamate receptor subunits in frontal cortex brain lysates from control subjects (*n*=5) and *PARK2* patients (*n*=4). No GluK1 expression was detectable. Results derive from three independent experiments. The densitometer analysis shows that GluK2 protein is significantly increased in brain tissues from patients with *PARK2* mutations (unpaired *t*-test; ***P=0.0002; *t*=7.259) and that GluA1 decreases in brain tissues from patients with *PARK2* mutations (unpaired *t*-test; ***P=0.0001; *t*=7.826). Error bars indicate±s.e.m.

The parkin-Q311X transgenic model expresses a human parkin variant causing ARJP^11^, and it is the first parkin-based mouse model that exhibits a neurodegenerative phenotype *in vivo*^12^. To test the hypothesis that parkin regulates glutamate ionotropic receptors, we analysed NMDAR, AMPAR and KAR subunit levels in substantia nigra from parkin-Q311X mice and littermate age-matched controls (mouse genotyping is reported in Supplementary Fig. 1a). The levels of the KAR GluK2 subunit were significantly higher in lysates from parkin-Q311X mice than from control mice (mean GluK2 values: controls 1.00±0.09 versus *PARK2* 1.51±0.08, unpaired *t*-test **P=0.0010), whereas the levels of AMPAR (GluA1 and GluA2/3) and NMDAR subunits (GluN1 and GluN2B) were similar in the two groups (Fig. 1a; GluK2a antibody validation is reported in Supplementary Fig. 1b; full blotting images are reported in Supplementary Fig. 2). To investigate whether GluK2 protein accumulates in ARJP patients’ brains, we analysed NMDAR, AMPAR and KAR subunit levels in brain lysates from healthy controls (*n*=5) and patients with the *PARK2* mutation (*n*=4). Patient’s data are reported in Supplementary Table. 1. GluA1 subunit levels were lower in brain lysates from patients than in those from controls (mean GluA1 values: controls 1.00±0.01 versus *PARK2* 0.58±0.06, unpaired *t*-test ***P=0.0001; Fig. 1b). GluK2 subunit levels were markedly higher in brain lysates from patients than in those from controls (mean GluK2 values: controls 1.00±0.03 versus *PARK2* 2.41±0.22, unpaired *t*-test ***P=0.0002; Fig. 1b; full blotting images are reported in Supplementary Fig. 3). GluK2 protein levels therefore increased in two independent *PARK2* models, the difference suggesting that parkin may interact with and regulate GluK2 protein.

### Parkin interacts with the KAR GluK2 subunit

**Figure 2:**
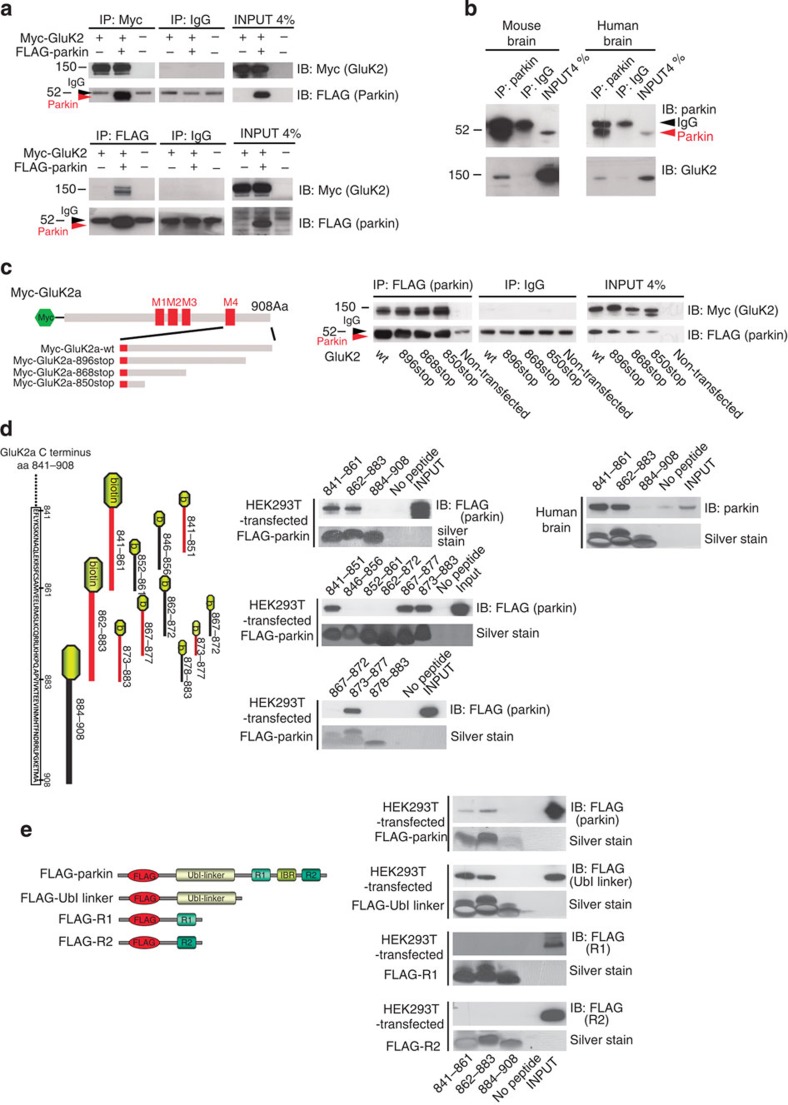
Parkin interacts with GluK2. (**a**) Representative western blottings of co-immunoprecipitations in HEK293T cells transfected with FLAG-parkin and Myc-GluK2a. The data show that FLAG-parkin co-immunoprecipitates with Myc-GluK2a and that Myc-GluK2a co-immunoprecipitates with FLAG-parkin. The image is representative of five independent experiments. (**b**) Representative western blottings of co-immunoprecipitations between endogenous parkin and GluK2 in whole mouse brain lysates and whole human brain lysates. The data show that GluK2 co-immunoprecipitates with parkin. The image is representative of three independent experiments. (**c**) Western blottings of co-immunoprecipitation experiments from HEK293T cells transfected with FLAG-parkin and Myc-GluK2a mutants truncated at aa 896, 868 and 850. Our terminology for these truncation mutants indicates the position at which a stop codon was introduced. All the mutants were able to bind parkin. The data show that the parkin-GluK2a interaction is unaffected by the loss of the last 58 aa of the GluK2a tail. The image is representative of four independent experiments. (**d**) Pull-down assay using biotinylated peptides spanning the GluK2a C terminus and lysates from human brain or HEK293T cells expressing FLAG-parkin. GluK2a peptides that bind parkin are indicated in red. The image is representative of five independent experiments. (**e**) Pull-down assay using biotinylated GluK2a peptides and lysates from HEK293T cells expressing FLAG-parkin or amino-terminally flagged parkin domains (FLAG-Ubl-linker; FLAG-R1; FLAG-R2). GluK2a peptides pulled down FLAG-Ubl-linker of parkin. The image is representative of five independent experiments.

We tested the interaction hypothesis in HEK293T cells transfected with GluK2a and parkin. We found that parkin co-immunoprecipitated with GluK2a and that GluK2a co-immunoprecipitated with parkin (Fig. 2a and Supplementary Fig. 4). The co-immunoprecipitation intensities were weak but consistently above background levels. Fluorescence resonance energy transfer (FRET) experiments between GluK2a-YFP and CFP-parkin expressed in HEK293T cells indicated a direct interaction between the two proteins (Supplementary Fig. 5a). We therefore hypothesized that the parkin-GluK2 interaction might be a transient phenomenon dependent on agonist stimulation. We found that activation of GluK2 KARs with 10 mM glutamate increased parkin-GluK2a interaction in HEK293T cells as assessed by immunoprecipitation (untreated 1.00±0.27 versus glutamate treated 2.70±0.31, unpaired *t*-test **P=0.0061; Supplementary Fig. 5b) and FRET experiments (Supplementary Fig. 5a).

We next investigated whether the parkin–GluK2 interaction exists in brain tissues, and found that endogenous GluK2 co-immunoprecipitated with parkin in lysates from wild-type (wt) mouse brains (Fig. 2b and Supplementary Fig. 6). No co-immunoprecipitation was detected in the negative controls, that is, lysates from parkin^−/−^ and GluK2^−/−^ mouse brains (Supplementary Fig. 5c). Finally, we showed that GluK2 co-immunoprecipitated with parkin in lysates from whole human brains (Fig. 2b and Supplementary Fig. 6). The co-immunoprecipitation intensities were weak but consistently above background levels. Hence, these data show that parkin interacts with GluK2.

Many GluK2 interactors bind to the GluK2 C-terminal cytoplasmic tail, which corresponds to amino acids (aa) 841–908 in the human GluK2a isoform^13^. We therefore conducted co-immunoprecipitation experiments between parkin and GluK2a mutants with truncations of the C-terminal tail. Parkin co-immunoprecipitated with all GluK2a mutants, including the shorter GluK2a-850stop (Fig. 2c and Supplementary Fig. 6), thus suggesting that parkin binds to aa 841–850 of GluK2a. A pull-down assay using biotinylated peptides spanning the GluK2a C terminus and lysates from human brain or HEK293T cells expressing FLAG-parkin showed that parkin binds to GluK2a aa 841–850 and that parkin can also bind to a second GluK2a domain lying between aa 873 and 877 (Fig. 2d and Supplementary Fig. 7). Once we had identified the GluK2a domain responsible for parkin binding, we investigated which parkin domain mediated parkin binding to GluK2. A pull-down assay using GluK2a peptides and parkin domains showed that parkin binding specifically depends on the Ubl-linker domain (Fig. 2e and Supplementary Fig. 7). Hence, data from Fig. 2 and Supplementary Figs 4–7 show that parkin interacts directly with GluK2 and that the interaction is mediated by the Ubl-linker domain of parkin and the proximal part of the GluK2 C-terminal cytoplasmic tail. In addition, glutamate stimulation of KARs favours the parkin–GluK2 interaction.

### Parkin can ubiquitinate GluK2

**Figure 3:**
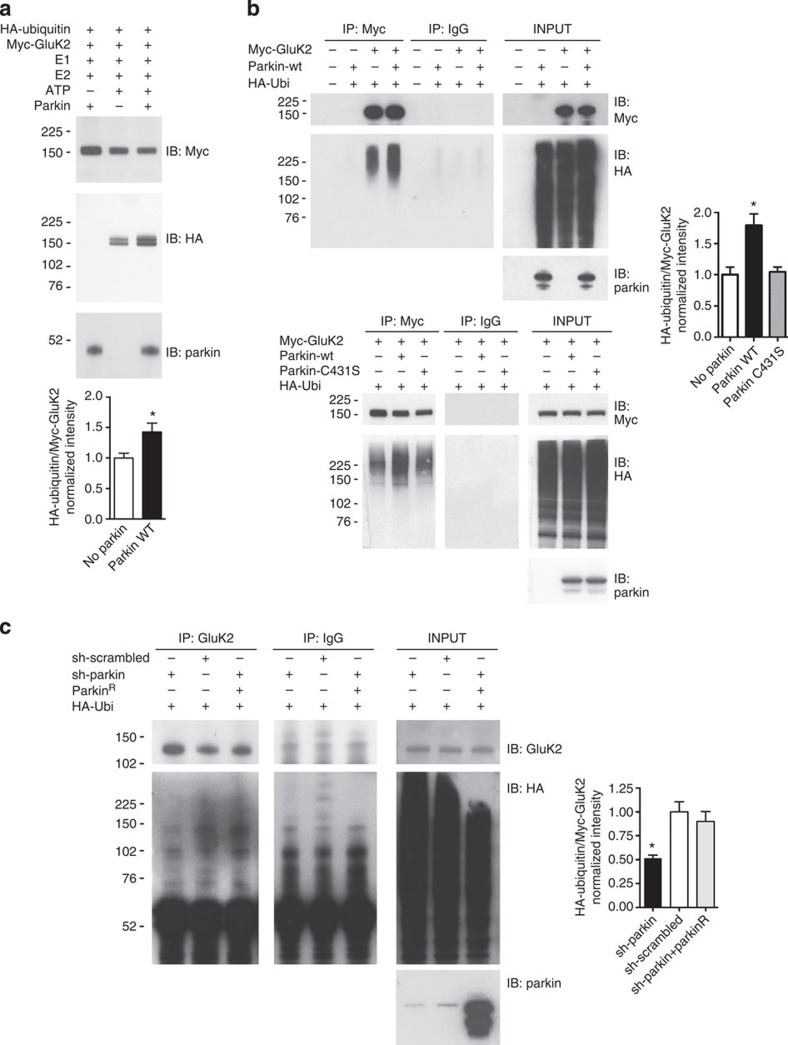
Parkin ubiquitinates GluK2. (**a**) *In vitro* ubiquitination assay of Myc-GluK2a using recombinant E1 (human ubiquitin-activating enzyme E1), recombinant E2 (UbcH7), HA-ubiquitin and parkin. Myc-GluK2a was slightly ubiquitinated in the presence of E1 and E2 alone but recombinant parkin significantly increased GluK2a ubiquitination. Results are representative of nine independent experiments (two-tailed unpaired *t*-test, *P=0.0201, *t*=2.580, df=16). Error bars indicate±s.e.m. (**b**) Western blotting of co-immunoprecipitation using HEK293T cells transfected with Myc-GluK2a, HA-ubiquitin, wt parkin or parkin C431S. GluK2a was ubiquitinated in HEK293T cells and parkin transfection significantly increased GluK2a ubiquitination. Parkin C431S transfection did not increase GluK2a ubiquitination. Results are representative of three independent experiments (one-way analysis of variance (ANOVA) and Tukey test, *P=0.0219, F=9.029). Error bars indicate±s.e.m. (**c**) Western blotting of co-immunoprecipitation using primary hippocampal neurons transfected with HA-ubiquitin and infected with lentiviral particle encoding sh-parkin or sh-scrambled, or sh-parkin+parkin^R^. Endogenous parkin silencing significantly decreased endogenous GluK2 ubiquitination. Parkin^R^ overexpression rescued endogenous GluK2 ubiquitination. The image is representative of three independent experiments that gave identical results (one-way ANOVA and Tukey test, *P=0.0169, F=8.689). Error bars indicate±s.e.m.

As parkin binding to GluK2 specifically depends on the Ubl-linker domain, which binds ubiquitination substrates^14^, we hypothesized that parkin could ubiquitinate GluK2. To test this possibility, we incubated purified GluK2a with HA-ubiquitin, E1, E2 (UbcH7) *in vitro*. We observed that recombinant parkin increased GluK2 ubiquitination (no parkin 1.00±0.08 versus wt parkin 1.43±0.14, unpaired *t*-test *P=0.0201; Fig. 3a and Supplementary Fig. 8). We confirmed that parkin-ubiquitinated GluK2a expressed in HEK293T cells transfected with Myc-GluK2a, HA-ubiquitin and parkin. Transfection of parkin significantly increased Myc-GluK2a ubiquitination, whereas transfection of the catalytically null mutant parkinC431S left GluK2a ubiquitination unchanged (controls 1.00±0.12 versus wt parkin 1.79±0.19 and parkinC431S 1.04±0.08, ANOVA *P=0.0219; Fig. 3b and Supplementary Fig. 8). To investigate whether parkin can ubiquitinate endogenous GluK2, we analysed GluK2 ubiquitination in primary hippocampal neurons. We silenced endogenous parkin in primary hippocampal neurons by lentiviral infection of a GFP-shRNA-parkin construct (Supplementary Fig. 9). In these experiments, we detected a ubiquitin smear in GluK2 immunoprecipitated samples; rat parkin silencing decreased GluK2 ubiquitination, whereas co-expression of shRNA-resistant human parkin (parkin^R^) increased GluK2 ubiquitination (sh-scrambled 1.00±0.10 versus sh-parkin 0.51±0.03 versus sh-parkin+parkin^R^ 0.90±0.10; ANOVA *P=0.0169; Fig. 3c and Supplementary Fig. 10). Hence, parkin ubiquitinates GluK2 *in vitro*, GluK2 expressed in heterologous cell cultures and GluK2 containing KARs in cultured hippocampal neurons. Knockdown of parkin function significantly decreases endogenous GluK2 ubiquitination.

### Loss of parkin increases KAR currents and excitotoxicity

**Figure 4:**
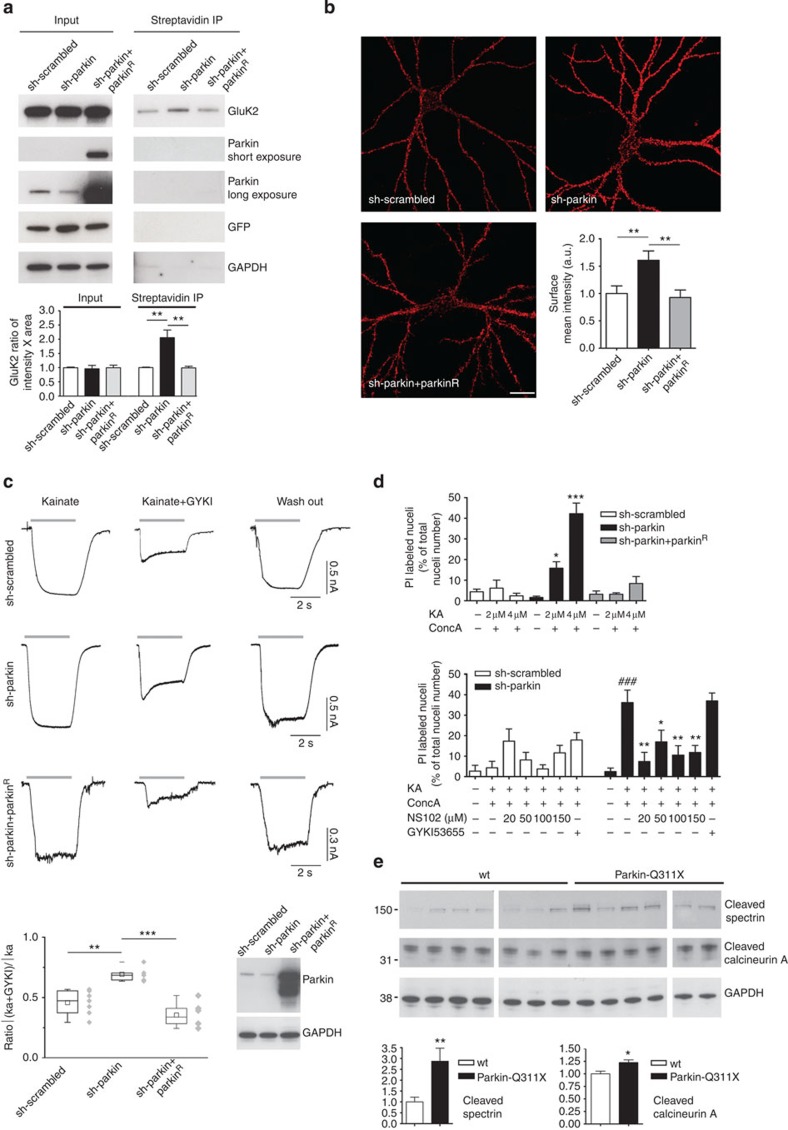
Loss of parkin function increases surface GluK2 levels, KAR currents and causes excitotoxicity. (**a**) Surface biotinylation assay in hippocampal neurons infected with lentivirus encoding sh-scrambled or sh-parkin, or sh-parkin+parkin^R^. Endogenous parkin silencing increased surface GluK2 levels; co-infection with lentivirus encoding parkin^R^ rescued GluK2 surface levels (one-way analysis of variance (ANOVA)–Dunnett’s multiple comparison test, **P=0.0053, F=14.25). (**b**) Surface Myc labelling in hippocampal neurons co-transfected with sh-parkin+Myc-GluK2a or sh-scrambled+Myc-GluK2a, or sh-parkin+parkin^R^+Myc-GluK2a (one-way ANOVA–Bonferroni test, **P=0.0066, F=6.082, 29 degrees of freedom; ten neurons analysed/each condition). Scale bar, 20 μm. (**c**) KAR current analysis in hippocampal neurons infected with lentivirus encoding GFP bicistronic sh-scrambled or sh-parkin, or sh-parkin+parkin^R^. Whole-cell responses were induced by rapid application of 100 μM kainate. To isolate KAR currents from those resulting from AMPAR opening, we added 10 μM GYKI 53655 (IC_50_=0.9±0.08 μM)^16^. Responses are shown before application (left panel), during the concomitant application of GYKI53655 (central panel) and after GYKI53655 washout (right panel). Western blotting shows parkin expression in the three experimental conditions. The chart plot shows the ratio between current stimulated by kainate+GYKI53655 and the current triggered by 3 s application of kainate alone (one-way ANOVA and Tukey test, **P<0.01; ***P<0.001; F=15.87). Error bars indicate 25th and 75th percentiles. (**d**) Primary hippocampal neurons were infected with lentivirus encoding GFP bicistronic sh-scrambled or sh-parkin, or sh-parkin+parkin^R^. Data represent the percentage of infected cells (green fluorescent cells) labelled by propidium iodide (PI). Kainate 2–4 μM or concanavalin A 200 μg ml^−1^ alone did not induce cell death. Kainate 2–4 μM+concanavalin A 200 μg ml^−1^ caused excitotoxicity in parkin-silenced cells. Co-infection with lentivirus encoding parkin^R^ rescued cell death (one-way ANOVA–Tukey test, *P<0.05 versus sh-scrambled 2 μM kainate; ***P<0.001 versus sh-scrambled 4 μM kainate; F=20.02). NS102 20–150 μM blocked excitotoxicity in parkin-silenced neurons (one-way ANOVA–Dunnett’s multiple comparison test, *P<0.05, **P<0.01, versus kainate treated; ^###^*P*<0.001 versus untreated, F=7.268). GYKI53655 (10 μM) did not rescue cell death. (**e**) Western blottings for cleaved spectrin and cleaved calcineurin A in substantia nigra lysates from 3-week-old controls (*n*=7) and littermate parkin-Q311X mice (*n*=6). Results derive from three independent experiments (unpaired *t*-test, **P=0.0096, *t*=3.128 for cleaved spectrin; *P=0.0124, *t*=2.941 for cleaved calcineurin A). All error bars in Fig. 4 histograms indicate±s.e.m.

Given that KAR surface levels are regulated by GluK2 conjugation to small ubiquitin-like modifier protein-1 (ref. 15), we hypothesized that GluK2 ubiquitination by parkin could regulate KAR surface levels. We silenced endogenous parkin in primary hippocampal neurons by lentiviral infection with GFP-shRNA-parkin construct (Supplementary Fig. 9). We found that parkin silencing specifically caused accumulation of endogenous GluK2 in the plasma membrane, whereas parkin^R^ co-expression led to the opposite effect (sh-scrambled 1.000±0.017 versus sh-parkin 2.053±0.274 versus sh-parkin+ parkin^R^ 0.991±0.0572, ANOVA **P=0.0053; Fig. 4a and Supplementary Fig. 11). In addition, we co-transfected neurons with sh-parkin and Myc-GluK2a, and confirmed by immunofluorescence that parkin silencing increased surface Myc-GluK2a (sh-scrambled 1.00±0.14 versus sh-parkin 1.61±0.17; sh-parkin+parkin^R^ 0.93±0.14; ANOVA **P=0.0066; Fig. 4b).

Loss of parkin function was reported to enhance glutamatergic synapse efficacy^6^. Here we tested a specific regulation of KAR function by parkin. We recorded currents evoked by kainate application in cultured hippocampal neurons. To isolate KAR-mediated currents evoked by kainate, we used GYKI 53655 (10 μM), a non-competitive AMPAR antagonist that does not affect KARs^16^. KAR-mediated currents evoked in hippocampal neurons expressing sh-parkin had higher amplitude than those evoked in neurons expressing sh-scrambled. Co-transfection with parkin^R^ decreased KAR-mediated currents (ratio between current intensity on kainate+GYKI 53655/kainate alone: sh-scrambled 0.4579±0.03769; sh-parkin 0.6790±0.03642; sh-parkin+parkin^R^ 0.3555±0.04194; ANOVA **P<0.01 sh-scrambled versus sh-parkin; ANOVA ***P<0.001 sh-parkin versus sh-parkin+parkin^R^; Fig. 4c). These data show that loss of parkin function increases the level of endogenous KARs, thus resulting in increased KAR currents evoked by kainate.

We next tested whether the KAR currents increase resulting from the loss of endogenous parkin increased neuron vulnerability to excitotoxicity. We measured cell death rates in primary hippocampal neurons treated with moderate kainate concentrations (2–4 μM) plus concanavalin A (200 μg ml^−1^), a lectin that specifically blocks KAR desensitization. Kainate plus concanavalin A induced dose-dependent cell death selectively in sh-parkin neurons (kainate 2 μM+ConcA 200 μg ml^−1^: sh-scrambled 6.15±3.90 versus sh-parkin 15.80±3.12, ANOVA *P<0.05; kainate 4 μM: sh-scrambled 2.40±1.29 versus sh-parkin 42.21±5.20, ANOVA ***P<0.001). Co-transfection with parkin^R^ rescued neuronal cell death (kainate 2 μM+ConcA 200 μg ml^−1^: sh-parkin+parkin^R^ 3.20±1.56; kainate 4 μM+ ConcA 200 μg ml^−1^: sh-parkin+parkin^R^ 8.39±3.38). Co-treatment with KAR antagonist NS102 (ref. 17) (20–150 μM) rescued cell death (value for sh-parkin: kainate 4 μM+ConcA 200 μg ml^−1^, 36.20±6.07 versus untreated 2.56±1.73, ANOVA ^###^*P*<0.001; kainate 4 μM+ConcA 200 μg ml^−1^+NS102 20 μM 7.46±4.44, ANOVA **P<0.01 versus kainate 4 μM+ConcA200 μg ml^−1^; kainate 4 μM+ConcA 200 μg ml^−1^+NS102 50 μM 17.01±5.72, ANOVA *P<0.05 versus kainate 4 μM+ConcA 200 μg ml^−1^; kainate 4 μM+ConcA 200 μg ml^−1^+NS102 100 μM 10.58±4.57, ANOVA **P<0.01 versus kainate 4 μM+ConcA200 μg ml^−1^; kainate 4 μM+ConcA 200 μg ml^−1^+NS102 150 μM 11.84±3.41 ANOVA **P<0.01 versus kainate 4 μM+ConcA200 μg ml^−1^). Co-treatment with the AMPAR antagonist GYKI53655 did not rescue cell death (value for sh-parkin: kainate 4 μM+ConcA 200 μg ml^−1^+GYKI53655 10 μM 37.05±3.80, ANOVA *P*>0.05 versus kainate 4 μM+ConcA200 μg ml^−1^; Fig. 4d). Hence, these data show that loss of parkin function increases the vulnerability of hippocampal neurons to KAR-dependent excitotoxicity.

To test whether the increased GluK2 levels were associated with increased vulnerability to excitotoxicity *in vivo*, we analysed neuronal levels of cleaved spectrin (non-erythroid alpha II-spectrin) and cleaved calcineurin A, both well-recognized markers for excitotoxic damage^18^,^19^,^20^. Cleaved spectrin and cleaved calcineurin A were significantly higher in substantia nigra of parkin-Q311X mice than in littermate controls (cleaved spectrin: wt 1.00±0.22 versus parkin-Q311X 2.87±0.60, unpaired *t*-test **P=0.0096; cleaved calcineurin A: wt 1.00±0.05 versus parkin-Q311X 1.23±0.05, unpaired *t*-test *P=0.0124; Fig. 4e and Supplementary Fig. 12). Hence, the increased GluK2 levels and excitotoxicity markers in our experiments disclosed in substantia nigra of parkin-Q311X mice suggest that KAR upregulation induces excitotoxicity *in vivo*.

## Discussion

In this study, we describe major new findings that extend our current knowledge about the molecular mechanism through which parkin regulates glutamatergic synapses^6^. Parkin interacts with and ubiquitinates the GluK2 KAR subunit and regulates GluK2 levels and KAR currents. A loss of parkin function increases KAR-dependent excitotoxicity *in vitro* and mutant parkin expression correlates with GluK2 accumulation and excitotoxicity features *in vivo*. Besides showing that parkin regulates KAR currents, we specify that it does so by binding to the GluK2 C-terminal cytoplasmic tail. Parkin-GluK2 binding appears as a transient effect dependent on KAR activation by agonists. As parkin affects somatodendritic KAR currents, the parkin-GluK2 interaction probably takes place at post-synaptic sites. Given that parkin is also localized in axons^21^, we cannot exclude the possibility that parkin regulates pre-synaptic GluK2-containing KARs, which are involved in the facilitation of synaptic transmission^22^. GluK2a interacts with Ubl-linker domain of parkin, similar to other parkin substrates^23^. The accumulation of GluK2 we found in brains from patients with *PARK2* mutations and in the *PARK2* mouse model parkin-Q311X further indicates that GluK2 is a ‘genuine’ parkin substrate. By identifying GluK2 as a parkin substrate, our results add to the evidence that parkin can ubiquitinate synaptic proteins^8^,^24^,^25^. As parkin activation is a multistep process involving relocalization and release of ubiquitination activity, which are probably regulated through phosphorylation or cysteine modification, or ligand binding to the Ubl domain^26^,^27^, we can speculate that GluK2 binding to Ubl domain may induce parkin activation.

The loss of parkin function increases surface and total GluK2 levels, and consistently increases KAR currents. These data agree with previous findings demonstrating that a loss of endogenous parkin increases excitatory synaptic transmission^6^. By identifying KAR as a direct target of parkin, our results provide a step ahead towards understanding the mechanism through which parkin modulates synaptic functions.

Our molecular findings also provide new insights into the mechanisms responsible for neurodegeneration in patients with ARJP. Similar to sporadic Parkinson’s disease, ARJP typically causes massive dopaminergic neuronal depletion in the substantia nigra pars compacta (SNc)^28^, a brain region receiving glutamatergic inputs from cortex, pedunculopontine nuclei^29^, superior colliculus^30^, thalamus^31^ and subthalamic nucleus (STN). These brain anatomical connections make the SNc neurons highly vulnerable to glutamate and suggested the longstanding hypothesis that SNc neuron depletion in Parkinson’s disease may stem from excitotoxicity^32^,^33^. SNc neurons abundantly express KAR subunits^34^. Although KAR function in the SNc remains elusive, our findings, showing that a loss of parkin function increases KAR function, support the excitotoxicity hypothesis in parkin-related ARJP. Current knowledge shows that in parkinsonian patients’ brain, the STN displays irregular burst firing deriving from dopamine deficiency^32^, resulting in high release of glutamate in the SNc, thereby causing excitotoxic damage^33^,^35^. In this context, a parkin loss of function may cause primary excitotoxic damage that would transiently precede the excitotoxic damage, depending on the overactivity in the STN glutamatergic neurons.

Hence, our results, suggesting that parkin modulates excitatory currents by binding to and promoting GluK2 ubiquitination, support the hypothesis that a loss of neuronal parkin increases vulnerability to excitotoxicity^6^ and suggest that patients with the *PARK2* mutation might benefit from neuroprotective therapy targeting KAR.

## Methods

### Glutamate receptor subunit analysis in mouse brain tissues

Parkin-Q311X (A) mice and littermate controls (all mice were female FVB/N strain) were purchased from Charles River (Calco, Italy). Mice were kept under environmentally controlled conditions on a 12-h light/dark cycle with food and water *ad libitum*. Three- to four-week-old mice were killed and the substantia nigra were immediately dissected out on ice. Tissues were homogenized with lysis buffer (20 mM Tris, pH 7.5, 150 mM NaCl, 1 mM EDTA, 1 mM EGTA, 1% Triton X-100, protease inhibitors (Roche), 50 μM MG132 and 10 mM N-ethylmaleimide (Sigma)). Western blottings were performed with Novex NuPAGE SDS–PAGE gels (Invitrogen). The following antibodies were used: GluA1 (13185, Cell Signaling, 1:500), GluA2/3 (AB1506, Millipore, 1:1,000), GluN2B (PA5-18536, Thermo Scientific, 1:300), GluK2 (TA310550, Origene,1:1,000) and GluK3 (H0002899, Abnova, 1:2,000), GAPDH (sc-25778, Santa Cruz, 1:1,000), Calcineurin A (ADI-SPA-610, Enzo Life Sciences 1:1,000) and Spectrin (2122, Cell Signaling, 1:1,000). The study was approved by The Ethics Committees of IRCCS Istituto Auxologico Italiano ``Comitato Etico dell'IRCCS Istituto Auxologico Italiano di Milano''. Experimental procedures took place in accordance with the European Communities Council Directive (86/809/EEC).

### Glutamate receptor subunit analysis in human brain tissues

Control and *PARK2* post-mortem brain tissues were from the Department of Neurology, Juntendo University School of Medicine. The study protocol was approved by the Human Ethics Review Committee at Juntendo University School of Medicine. Informed consent to use human tissues were obtained from patients or close family members. Patients’ data are reported in Supplementary Table 1. Control brain tissues were autopsied brain tissue from age-matched controls who had undergone neuropathological examination to exclude neurodegenerative disorders. Tissues were homogenized with lysis buffer (20 mM Tris, pH 7.5, 150 mM NaCl, 1 mM EDTA, 1 mM EGTA, 1% Triton X-100, protease inhibitors (Roche), 50 μM MG132 and 10 mM N-ethylmaleimide (Sigma)). Western blottings were performed with Novex NuPAGE SDS–PAGE gels (Invitrogen). The following antibodies were used: GluA1 ((13185, Cell Signaling, 1:500), GluA2/3 (AB1506, Millipore, 1:1,000), GluN1 (clone N308/48, NeuroMab, 1:300), GluN2B (PA5-18536, Thermo Scientific, 1:300), GluK1 and GluK3 (H0002897 and H0002899, respectively, Abnova, 1:2,000), GluK2 (TA310550, Origene, 1:1,000), parkin (P6248, Sigma, 1:6,000), GAPDH (sc-25778, Santa Cruz, 1:1,000).

### Analysis of GluK2–parkin interaction

Plasmids encoding complementary DNA for human parkin and parkin domains (Ubl-linker, aa 1–237; RING1, aa 217–310; RING2, aa 395–465) were described previously^36^. Plasmids encoding truncated GluK2 mutants were created by mutagenizing the Myc-GluK2a(R) plasmid^37^ using the QuikChange mutagenesis kit (Stratagene). Samples were immunoprecipitated as described^38^ according to the following protocol: 500 μg of sample were pre-cleared by adding 50 μl of washed Protein A or G agarose bead slurry (catalogue number 17-5138-01 and 17-0618-01, GE Healthcare). The agarose beads were collected by pulsing 5 s in the microcentrifuge at 14,000 *g* and discarded. Sample supernatants were incubated with Myc antibody (R950, Invitrogen, 1:500) or parkin antibody (P6248, Sigma, 1:1,000), or FLAG antibody (M2, Sigma, 1:500) at 4 °C overnight. Fifty microlitres of washed Protein A or G agarose bead slurry were added to the reaction mixture and gently rocked at 4 °C for additional 2 h. The agarose beads were then collected by pulsing 5 s in the microcentrifuge at 14,000 *g* and washed three times with ice-cold washing buffer (20 mM Tris, pH 7.5, 500 mM NaCl, 1 mM EDTA, 1 mM EGTA, 1% Triton X-100, protease inhibitors (Roche), 50 μM MG132). The beads were resuspended in 40 μl of 2 × Laemmli sample buffer, boiled, analysed by SDS–PAGE with Novex NuPAGE SDS–PAGE gels (Invitrogen).

For pull-down experiments, lysates (150 μg) were incubated overnight at 4 °C with 15 μg of biotinylated peptide (Primm srl) previously conjugated to streptavidin-conjugated magnetic beads (Invitrogen), washed in lysis buffer, resuspended in SDS sample buffer and boiled. Gels were silver-stained with the SilverQuest Silver Staining Kit (Invitrogen). Parkin was labelled by parkin antibody (P6248, Sigma) or FLAG antibody (M2, Sigma).

### FRET analysis

Parkin cDNA was subcloned into a pECFP-C1 vector (Clontech). The plasmid encoding GluK2a C-terminally conjugated to enhanced yellow fluorescence protein (YFP) has been previously described^39^. FRET videos were acquired with an AF6000 microscope (Leica) equipped with a FRET excitation and emission fast filter wheel and a × 100 oil objective at a rate of one frame per minute. Each frame was acquired on three channels: CFP excitation and CFP emission (CFP), CFP excitation and YFP emission (FRET), and YFP excitation and YFP emission (YFP). FRET efficiency was calculated pixel*-*by-pixel according to the following formula (1):


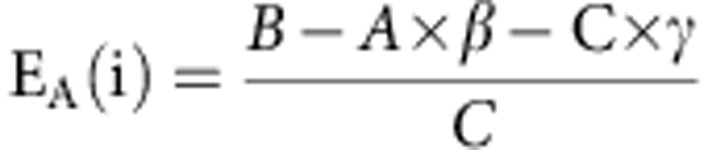


where *A*, is the fluorescence intensity measured in the CFP channel, and *B* and *C* are the fluorescence intensities measured in the FRET and YFP channels, whereas *β* and *γ* are correction factors for the spectral bleed throughout our constructs (Leica LAS AF software method 1). FRET efficiency increase was calculated by comparing the FRET efficiency distribution for three frames before and after treating for 10 min with 10 mM glutamate.

### Ubiquitination assays

For *in vitro* ubiquitination assay, purified Myc-tagged GluK2 (1 μM, Origene) was incubated in ubiquitinylation assay buffer (10 × buffer, 5 mM Mg-ATP, 1 mM dithiothreitol and 20 U ml^−1^ pyrophosphatase; Biomol) together with 100 nM recombinant human ubiquitin-activating enzyme E1 (Biomol), 2.5 μM recombinant E2 UbcH7 (Biomol), 2.5 μM haemagglutinin (HA)-tagged ubiquitin and 100 nM recombinant His-tagged parkin (Boston Biochem) for 2 h at 37 °C. The samples were immunoprecipitated with Myc antibody (R950, Invitrogen) and analysed by western blotting.

For ubiquitination assay in HEK293T, cells were transfected with plasmid encoding human parkin wt, parkin C431S, MycGluK2a plasmid and HA-ubiquitin. Cells lysates were immunoprecipitated with Myc antibody (R950, Invitrogen). Immunoprecipitated samples were analysed by western blotting with horseradish peroxidase-conjugated antibody against Myc (R951-25, Invitrogen) and HA (130-091-972, Miltenyi Biotec).

For analysis of endogenous GluK2 ubiquitination in primary neurons, lysates were immunoprecipitated with anti GluK2/3 antibody (clone NL9 Millipore) according to the manufacturer’s protocol. Five hundred micrograms of sample were pre-cleared by adding 50 μl of washed Protein A agarose bead slurry (catalogue number 17-5138-01, GE Healthcare). The agarose beads were collected by pulsing 5 s in the microcentrifuge at 14,000 *g* and discarded. Sample supernatants were incubated with 7.5 μl of anti-GluK2/3 at 4 °C overnight. Fifty microlitres of washed Protein A agarose bead slurry were added to the reaction mixture and gently rocked at 4 °C for additional 2 h. The agarose beads were then collected by pulsing 5 s in the microcentrifuge at 14,000 *g* and washed three times with ice-cold washing buffer (20 mM Tris, pH 7.5, 500 mM NaCl, 1 mM EDTA, 1 mM EGTA, 1% Triton X-100, protease inhibitors (Roche), 50 μM MG132). The beads were resuspended in 40 μl of 2 × Laemmli sample buffer, boiled, analysed by SDS–PAGE with Novex NuPAGE SDS–PAGE gels (Invitrogen) and developed with an antibody against HA (H6908, Sigma, 1:1,000).

### Lentiviral infections in neurons

Primary rat hippocampal neurons were prepared from embryonic day 18–19 rat brains^38^ and plated on poly-D-lysine (30 μg ml^−1^)-coated coverslips at densities of 75,000 cells per well for immunochemistry, 300,000 cells per well for biochemistry experiments and 150,000 cells per well for electrophysiological experiments. Neurons were infected with lentivirus at Days in Vitro (DIV) 8 (ref. 40). The pRNAT plasmid encoding short hairpin RNA (shRNA) specific against rat parkin and scrambled have been previously described^6^: parkin shRNA sequence was 5′-CCAAACCGGATGAGTGGAGAGTGCCAATC-3′. The same shRNA sequences were subcloned into the pLVTHM plasmid and used to prepare lentiviral particles. Rescue experiments were performed by expression of human parkin cDNA.

### Biotinylation assay and immunofluorescence in neurons

Plasma membrane proteins were biotinylated using membrane-impermeant sulfo-NHS-SS-biotin (0.3 mg ml^−1^, Pierce) for 5 min at 37 °C. Labelled neurons were washed with TBS supplemented with 0.1 mM CaCl_2_, 1.0 mM MgCl_2_ and 50 mM glycine. After further washes with TBS supplemented with 0.1 mM CaCl_2_ and 1.0 mM MgCl_2_ on ice, the cells were lysed in extraction buffer (50 mM Tris-HCl, pH 7.4, 1 mM EDTA, 150 mM NaCl, 1% SDS and protease inhibitor mixture). Lysates were boiled for 5 min and immunoprecipitated with streptavidin-conjugated magnetic beads (Dynabeads, Invitrogen).

For immunofluorescence, neurons were transfected at DIV 13–14 with the Myc-GluK2a(R) plasmid using the calcium phosphate method^40^. For surface staining, live DIV 18 hippocampal neurons were incubated for 10 min at 37 °C with Myc antibody. After washing (PBS supplemented with 1 mM MgCl_2_ and 0.1 mM CaCl_2_), neurons were fixed for 7 min at room temperature in 4% paraformaldehyde/4% sucrose without permeabilization and stained with a Cy3-conjugated secondary antibody (Invitrogen) applied in gelatin detergent buffer (30 mM phosphate buffer at pH 7.4 containing 0.2% gelatin, 0.5% Triton X-100 and 0.8 M NaCl). Fluorescent images were acquired and quantified^40^: labelled transfected neurons were chosen randomly for quantification from four coverslips from five independent experiments for each construct. Fluorescence images were acquired with a Biorad MRC1024 confocal microscope, using a Nikon × 60 objective with sequential acquisition setting at 1,280 × 1,024 pixel resolution. All morphometric measurements were made with Metamorph image analysis software (Universal Imaging Corporation). Both image acquisition and morphometric quantification were performed by investigators who were ‘blind’ to the experimental condition.

### KAR current recording in *in vitro* neurons

Whole-cell patch-clamp data were recorded from DIV 19–21 primary hippocampal neurons (clamped at −70 mV). The coverslips were perfused with the following extracellular solution (mM): 140 NaCl, 3 KCl, 2 CaCl_2_, 1.2 MgCl_2_, 10 glucose and 10 HEPES (pH 7.4). Borosilicate glass pipettes (5–8 MΩ resistance) were filled with the following solution (mM): 126 K-gluconate, 4 NaCl, 10 HEPES, 10 glucose, 1 MgSO_4_, 0.5 CaCl_2_, 1 EGTA, 3 ATP-Mg and 0.1 GTP-Na (pH 7.2). Recordings were accepted for analysis when membrane resistance (Rm) was >150 MΩ, series resistance (Rs) was <15 MΩ and, together with membrane capacitance (Cm), they changed by no more than 10% from the original value during the recording. Holding currents were lower than −100 pA and remained significantly unchanged throughout the experiments (Rm=268±31 MΩ, Rs=9.1±0.6 MΩ, Cm=42.2±2.3 pF and holding current −39.0±5.2 pA). Signals were derived using a Multiclamp 700B amplifier (Axon Instruments), digitized through a Digidata 1440A interface (Axon Instruments) and acquired for online visualization and offline analysis using pClamp10 Software (Axon Instruments). Data were recorded at 28–30 °C using a TC-324B temperature control (Warner Instruments).

### Excitotoxicity assay

Neurons were infected at DIV 8 with green fluorescent protein (GFP) sh-scrambled or sh-parkin or sh-parkin+parkin^R^. One week after infection, neurons were treated as indicated in Fig. 4d. Eight hours after the treatment, neurons were washed in standard neurobasal medium and then incubated with 1 μg ml^−1^ of propidium iodide for 10 min. Coverslips were PBS washed, fixed with 4% paraformaldehyde for 10 min and mounted with ProLong mounting DAPI (4′,6-diamidino-2-phenylindole) (Invitrogen, P36931). Images were acquired at confocal microscopy (Nikon Eclipse C1) and neurons were counted in ten fields from each coverslip. The percentage of dead neurons was calculated as the ratio between red-labelled neurons (propidium iodide) and green fluorescent cells (GFP infected). Approximately 800 to 1,300 neurons were scored for each condition. Experiments were repeated three times each on three separate cultures.

### Statistical analysis

Statistical analyses were performed using GraphPad Prism4 software. Data were subjected to normality test (Kolmogorov–Smirnov test). Student’s *t*-test was used to compare two groups and one-way analysis of variance followed by appropriate *post-hoc* tests was used to compare multiple groups. Data are presented as mean±s.e.m.

## Author contributions

J.S. initiated the project and performed the parkin-GluK2 interaction experiments, which were further characterized by A.M. A.M. did the western blottings on human brain samples, the *in vivo* ubiquitination analysis and the biotinylation assay. A.M. performed the pull-down assay, the *in vitro* ubiquitination assay, the excitotoxicity experiments and mouse genotyping. A.F. conducted the fluorescence imaging experiments, prepared the lentiviral particles and performed neuron infections under the direction of M.P. F.S. performed the ubiquitination assay in HEK293T cells. G.R. conducted the FRET experiments under the direction of E.C. G.C. managed the Q311X mouse colony. Clinical samples and patient data were obtained by S.S. and N.H. M.M. performed the electrophysiology experiments. C.M. provided Myc-GluK2 plasmids, provided GluK2^−/−^ lysates and critically revised the manuscript. J.S. and A.C. conceived the study. A.M., A.C., M.P. and J.S. wrote the manuscript. All authors discussed the results, conceived further experiments, commented on the manuscript and approved the final submitted version.

## Additional information

**How to cite this article:** Maraschi, A. M. *et al.* Parkin regulates kainate receptors by interacting with the GluK2 subunit. *Nat. Commun.* 5:5182 doi: 10.1038/ncomms6182 (2014).

## Supplementary Material

Supplementary Information Supplementary Figures 1-12 and Supplementary Table 1
