# Supplementary material for: Parkin regulates kainate receptors by interacting with the GluK2 subunit
Source: Nat Commun. 2014 Oct 15;5:5182. doi: 10.1038/ncomms6182 (PMC4218952; doi:10.1038/ncomms6182)
Supplement: Supplementary Information — Supplementary Figures 1-12 and Supplementary Table 1 [file ncomms6182-s1.pdf]

Supplementary Figure 1

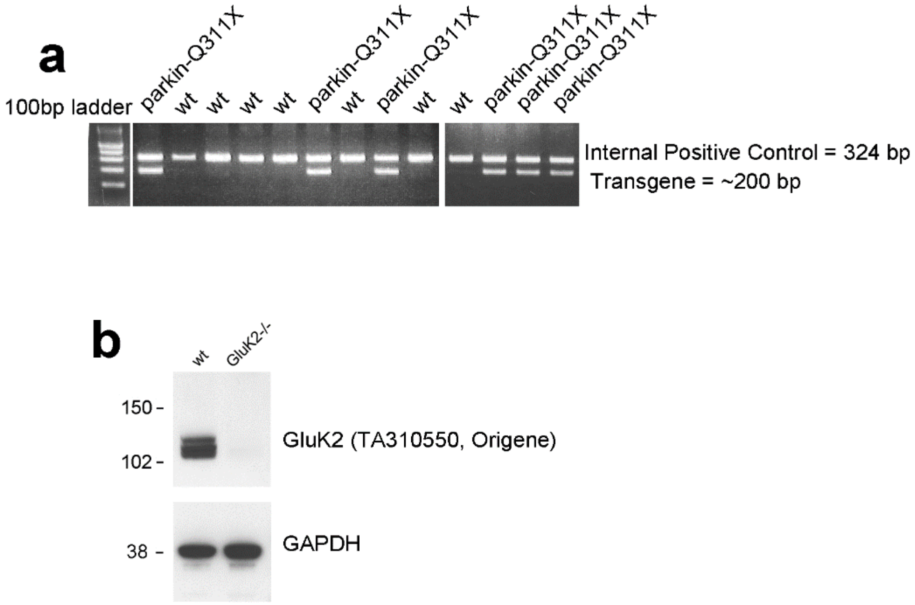

Supplementary Figure 1. Genotyping of parkin-Q311X mice and GluK2 antibody validation. (**a**) Genotyping of parkin-Q311X mice and littermate controls. The detection of the 324 bp band in all samples served as control for DNA quality and correct amplification. Band at ~200bp indicates the mutant *PARK2* transgene. **(b)** Whole brain lysates were prepared from wild-type and GluK2$^{-/-}$ mice. Western blot was performed with the antibody TA310550 (Origene). Antibody TA310550 specifically recognized GluK2.

Supplementary Figure 2

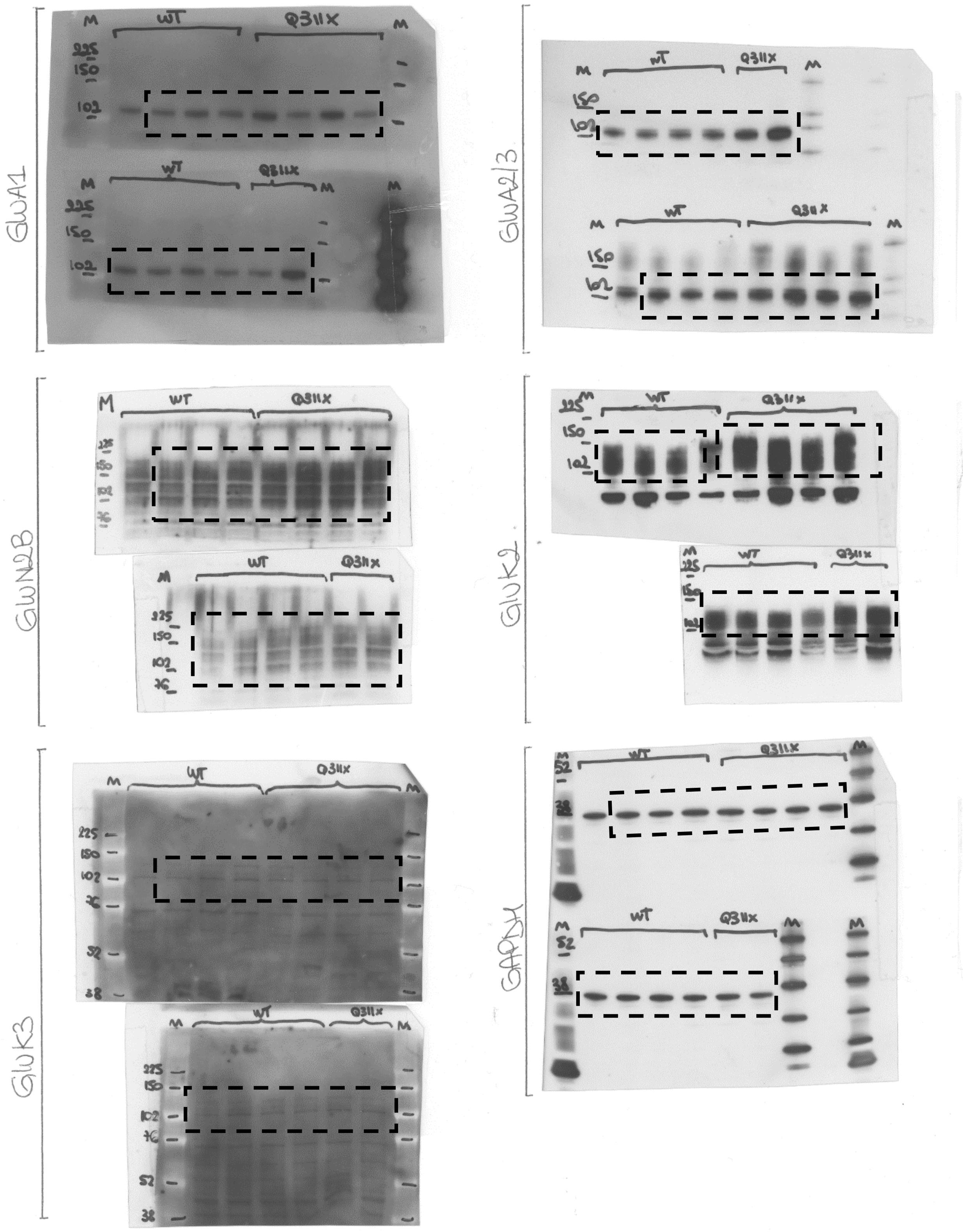

Supplementary Figure 2. Full blot images of western blots showed in Figure 1a.

Supplementary Figure 3

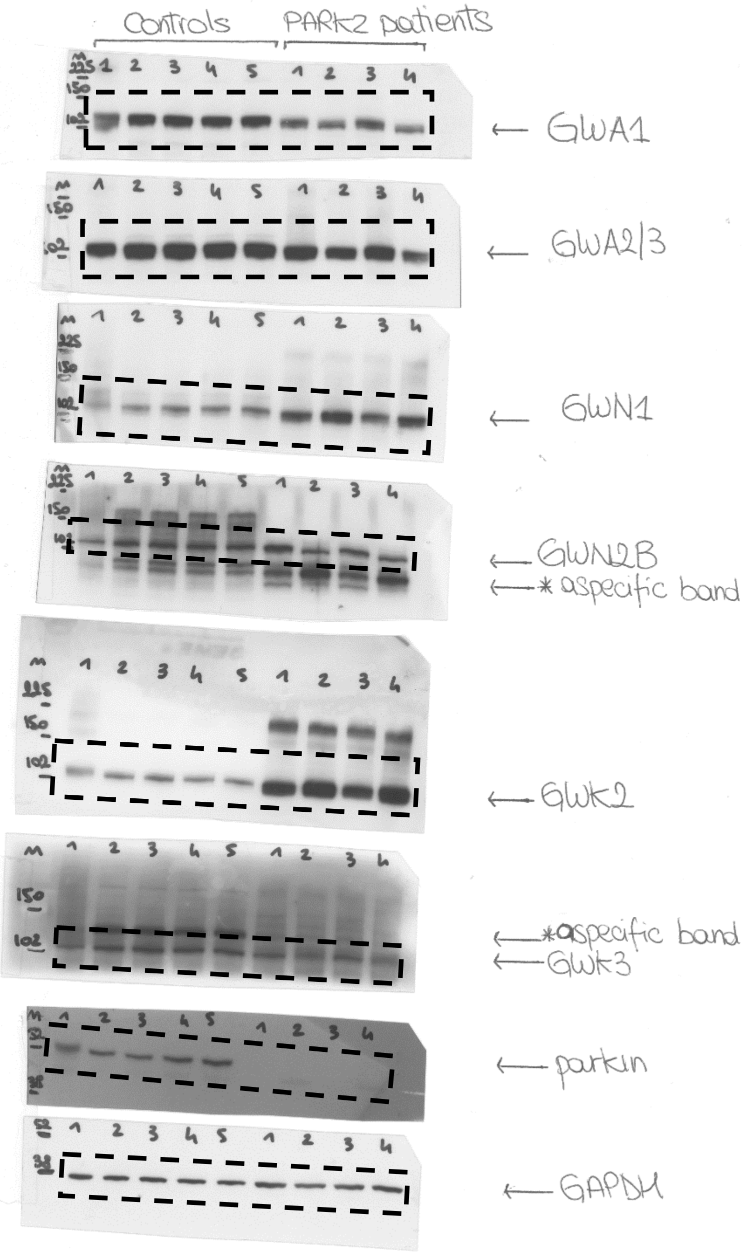

Supplementary Figure 3. Full blot images of western blots showed in Figure 1b.

Supplementary Figure 4

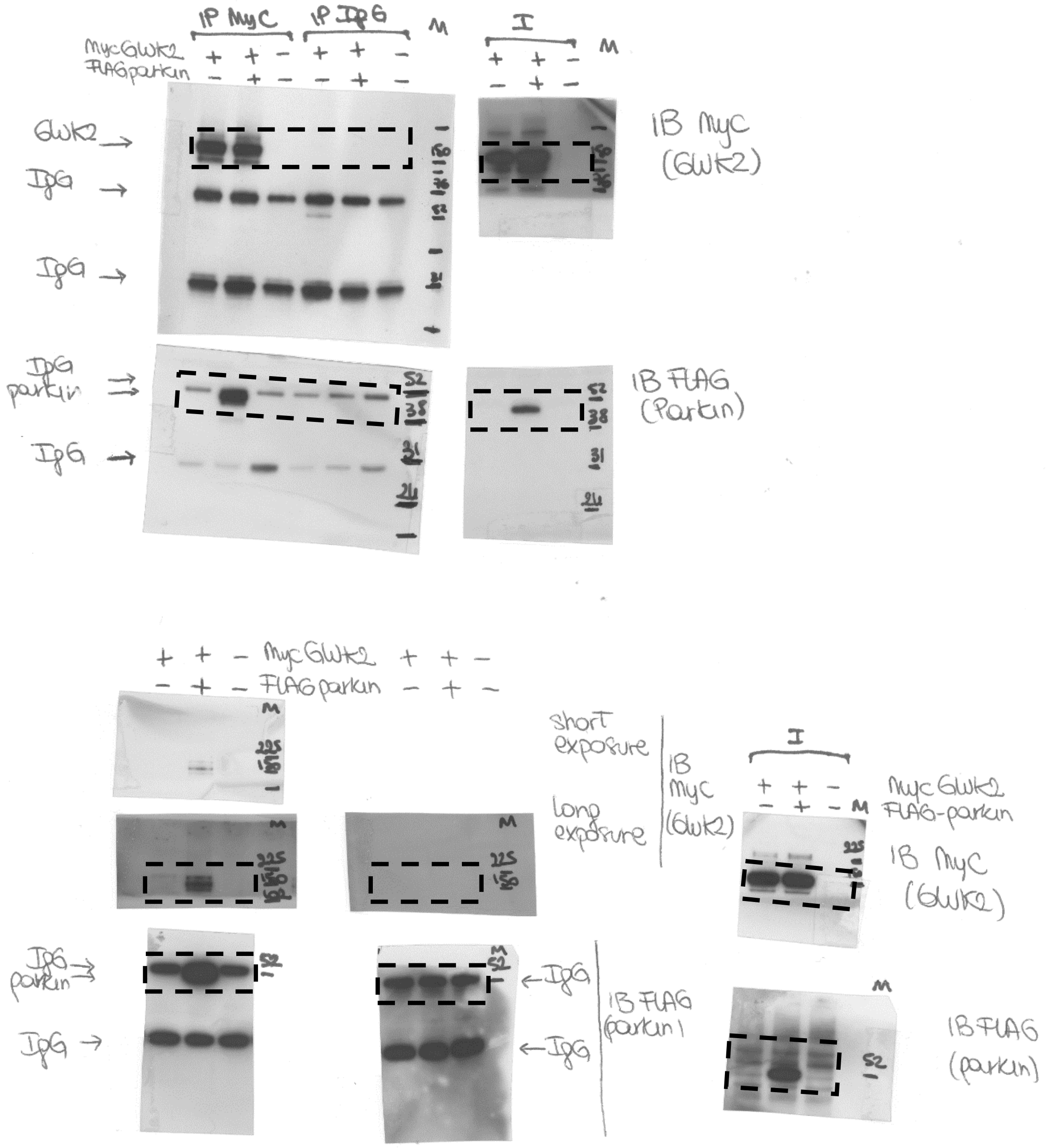

Supplementary Figure 4. Full blot images of western blots showed in Figure 2a.

Supplementary Figure 5

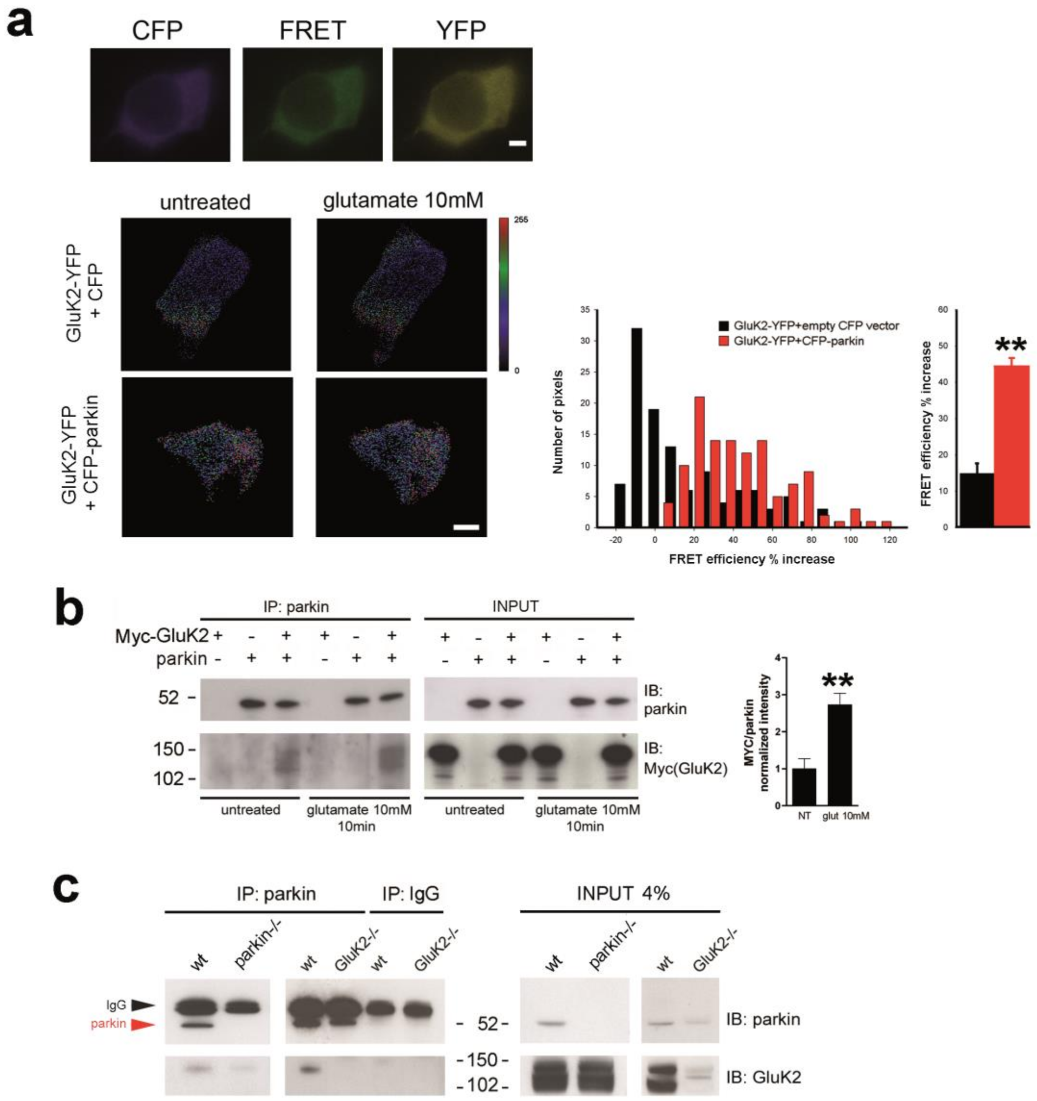

Supplementary Figure 5. KAR stimulation increases parkin-GluK2a interaction. (**a**) FRET analyses in HEK293T cells transfected with CFP-parkin and GluK2a-YFP. The graphs represent FRET efficiency increase upon glutamate treatment (Mann-Whitney Rank sum test, *P*=0.001, Degrees of freedom =117 Mann-Whitney U statistic = 2834; Bar is 10 μm). Error bars indicate±s.e.m. (**b**) Western blots of co-immunoprecipitation experiments from HEK293T cells transfected with parkin and Myc-GluK2a under basal condition and after treatment with 10 mM glutamate for 10 min. The histogram represents the densitometer values of samples transfected with parkin and Myc-GluK2a immunoprecipitated with parkin antibody. Data derive from four independent experiments (NT 1.00±0.27 vs glutamate treated 2.70 ± 0.31, two-tailed unpaired t test, ***P*=0.0061 t=4.1353 with 6 degrees of freedom). Error bars indicate±s.e.m. (**c**) Representative western blot of coimmunoprecipitations between endogenous parkin and GluK2 in brain lysates from wt-mouse, GluK2$^{-/-}$ mouse and parkin $^{-/-}$ mouse. The data show that GluK2 coimmunoprecipitates with parkin in wild-type tissues whereas coimmunoprecipitation signal is absent in lysates from parkin $^{-/-}$ mouse and GluK2$^{-/-}$ mouse. The image is representative of three independent experiments.

Supplementary Figure 6

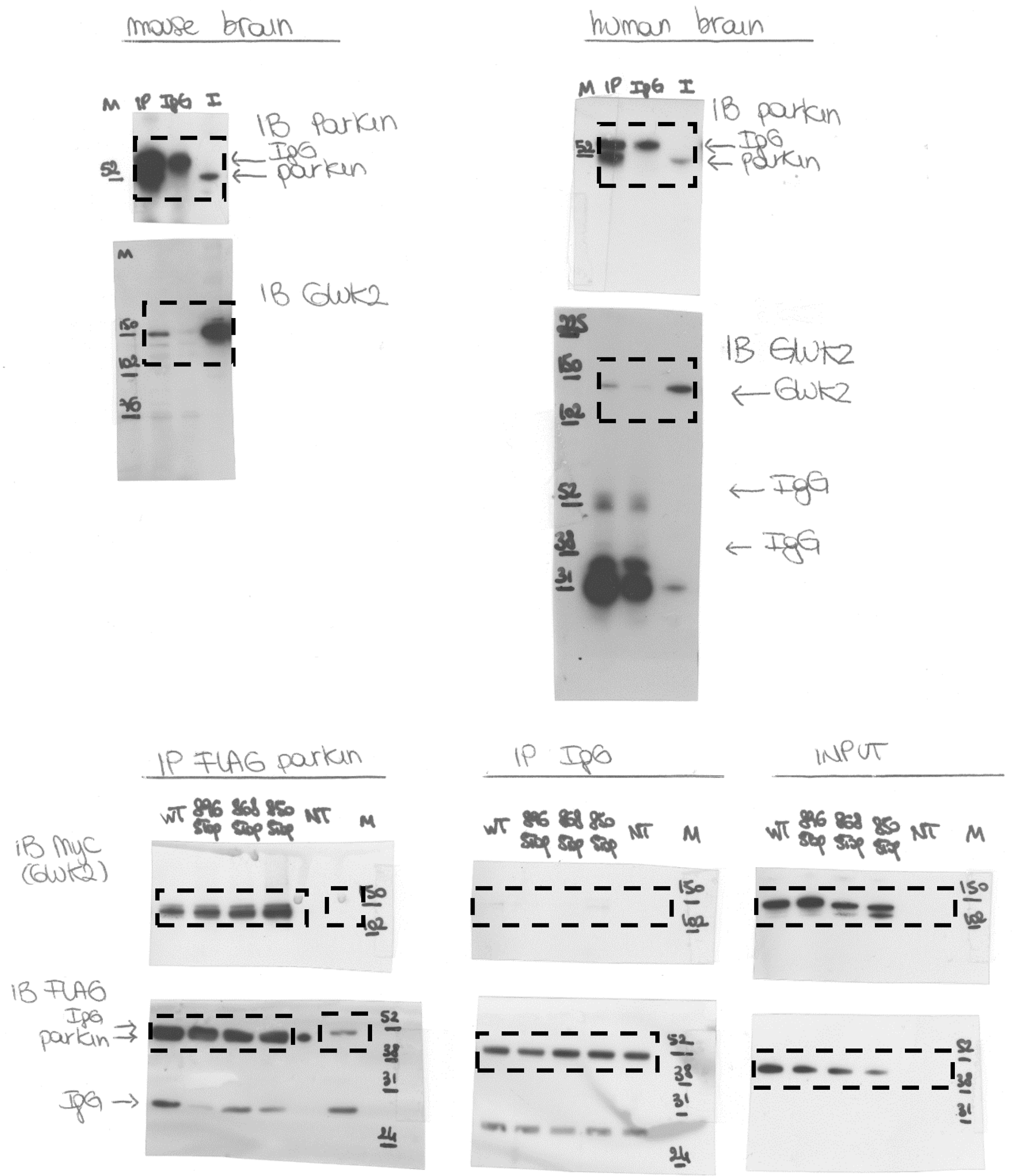

Supplementary Figure 6. Full blot images of western blots showed in Figure 2b-c.

Supplementary Figure 7

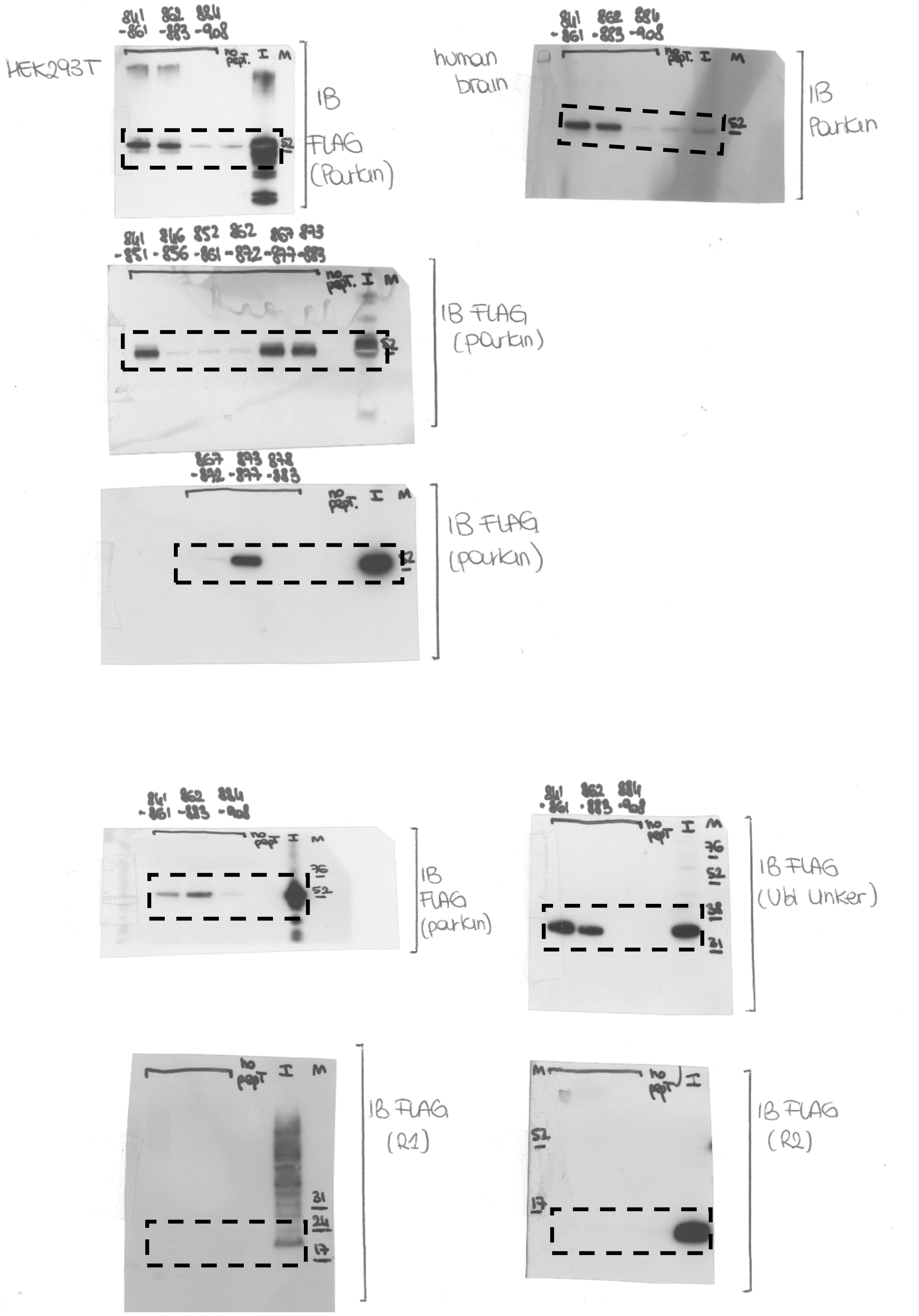

Supplementary Figure 7. Full blot images of western blots showed in Figure 2d-e.

Supplementary Figure 8

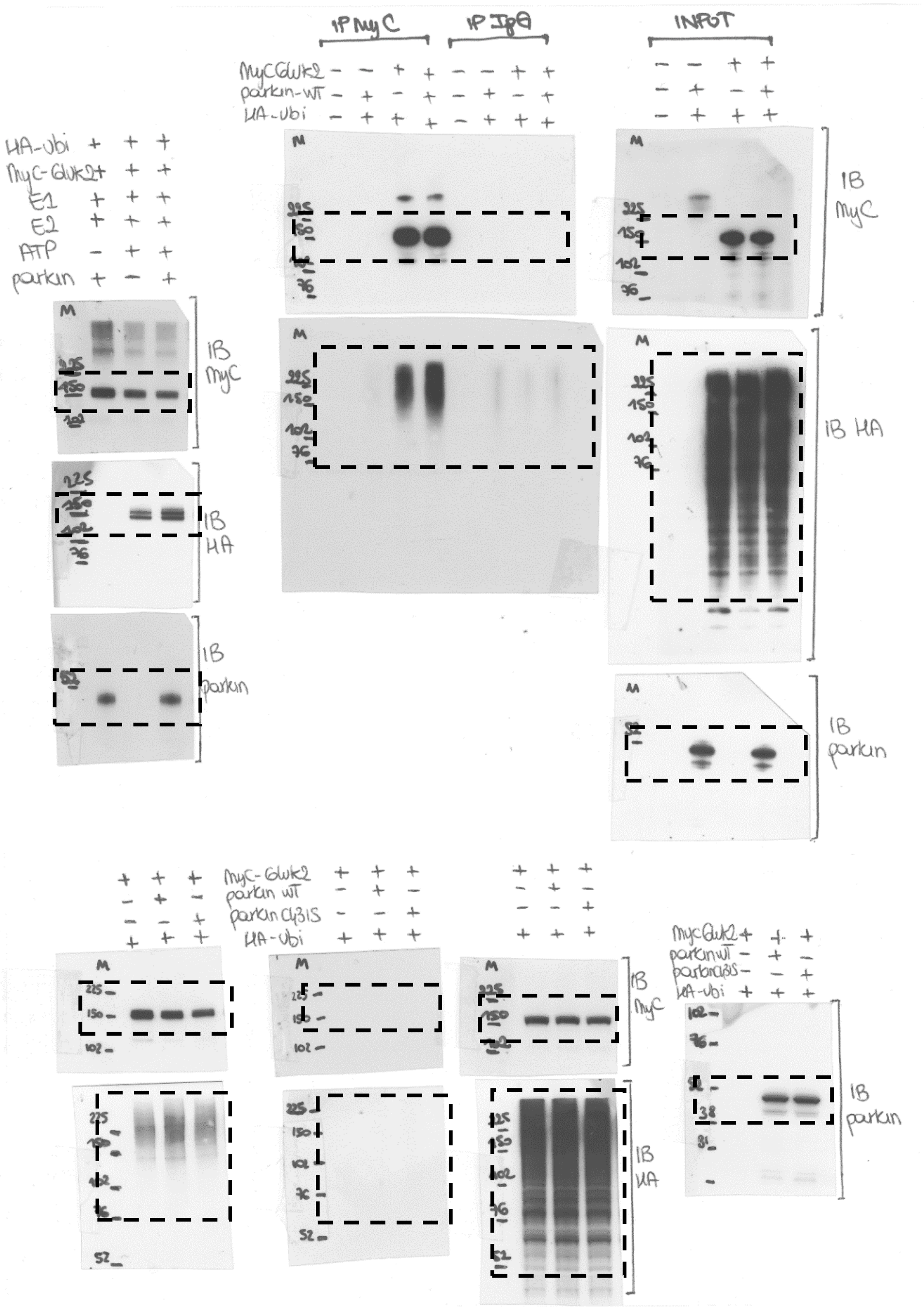

Supplementary Figure 8. Full blot images of western blots showed in Figure 3a-b.

Supplementary Figure 9

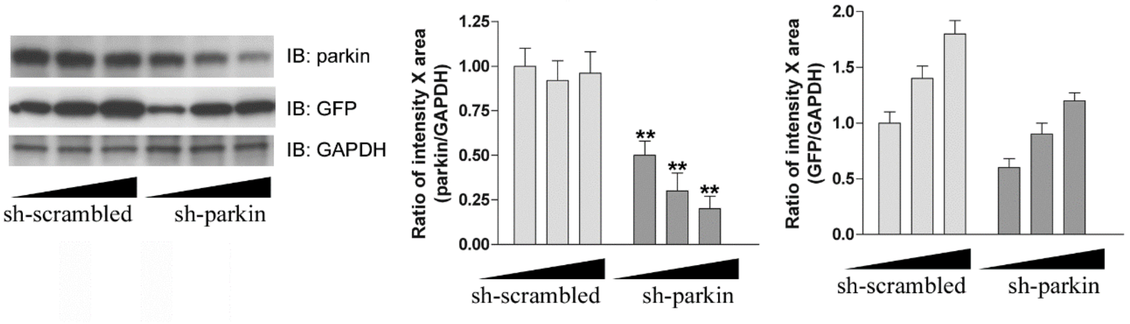

Supplementary Figure 9. Endogenous parkin silencing in primary neurons by lentivirus encoding short hairpin RNA. Western blot of total lysates from hippocampal neurons infected with increasing amounts (1, 2 and 3 μl/1.8 cm^2 well) of lentiviral particles encoding bicistronic GFP-shRNA-parkin or GFP-shRNA-scrambled. Densitometry analysis shows a dose-dependent decrease in parkin expression (***P*<0.01 vs scrambled) and a dose-dependent GFP expression increase. The image is representative of three independent experiments. Error bars indicate±s.e.m.

Supplementary Figure 10

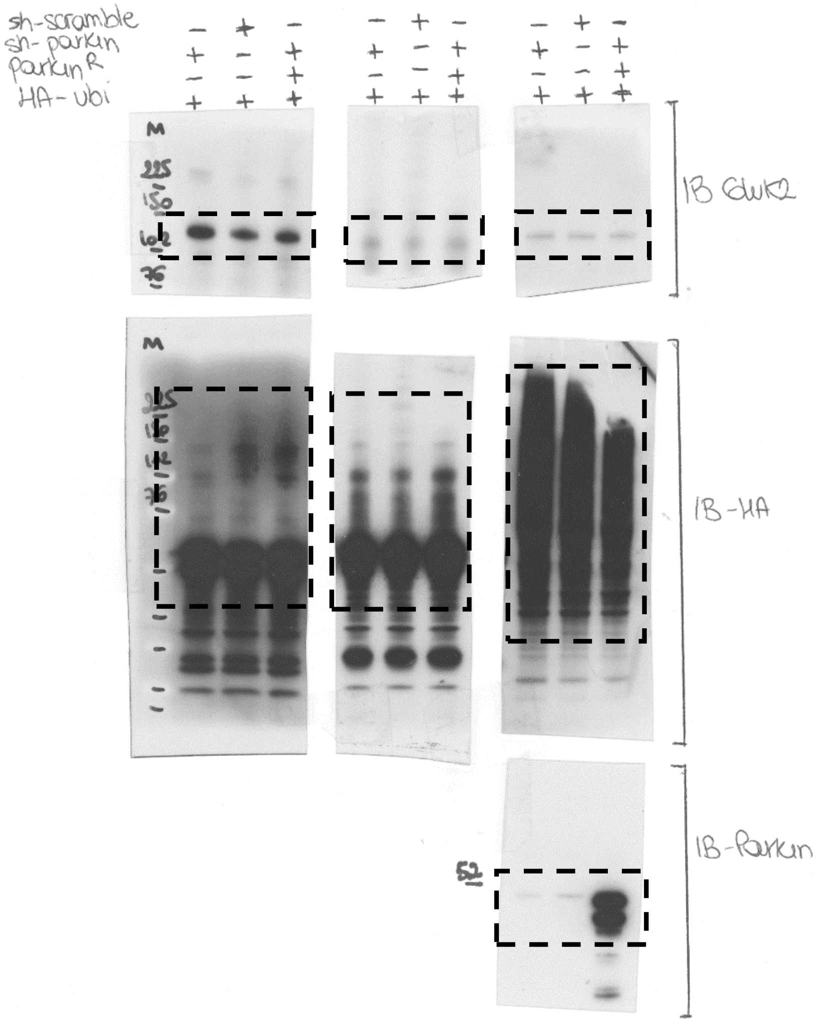

Supplementary Figure 10. Full blot images of western blots showed in Figure 3c.

Supplementary figure 11

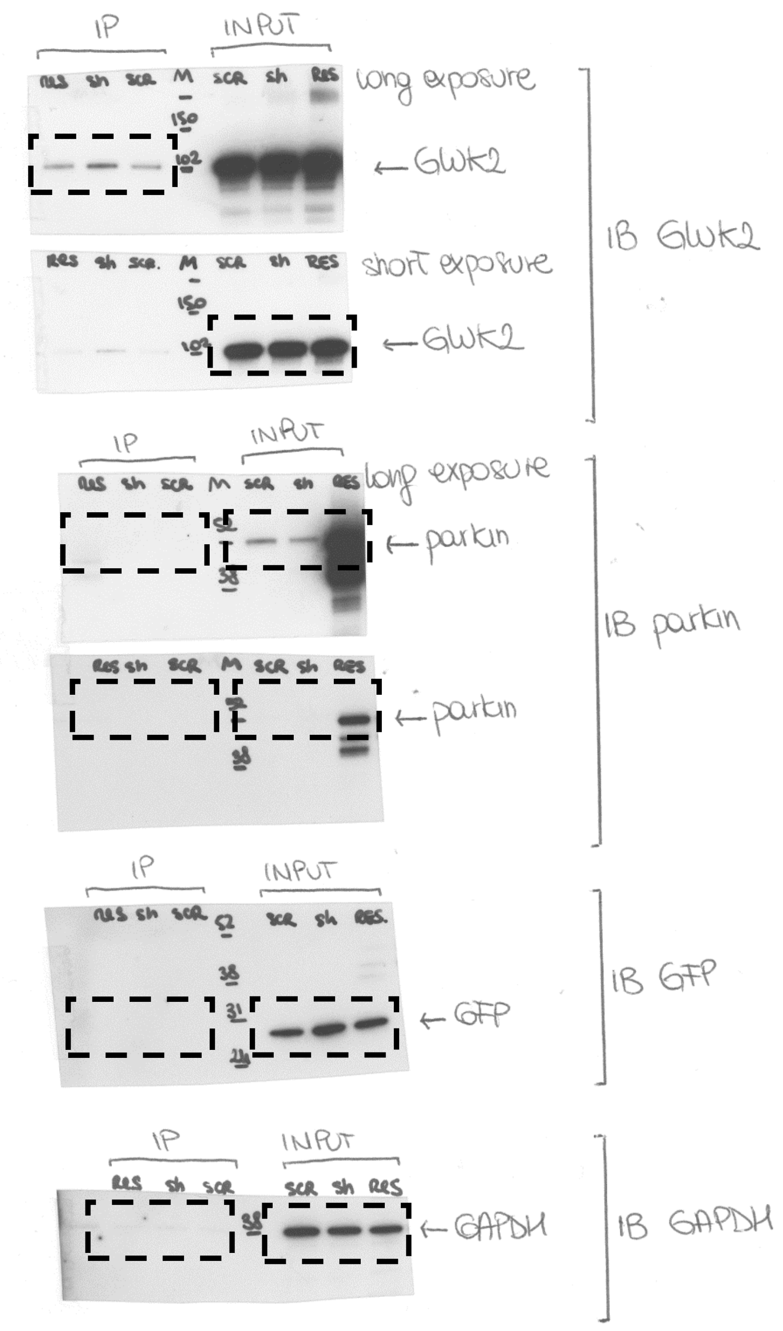

Supplementary Figure 11. Full blot images of western blots showed in Figure 4a.

Supplementary Figure 12

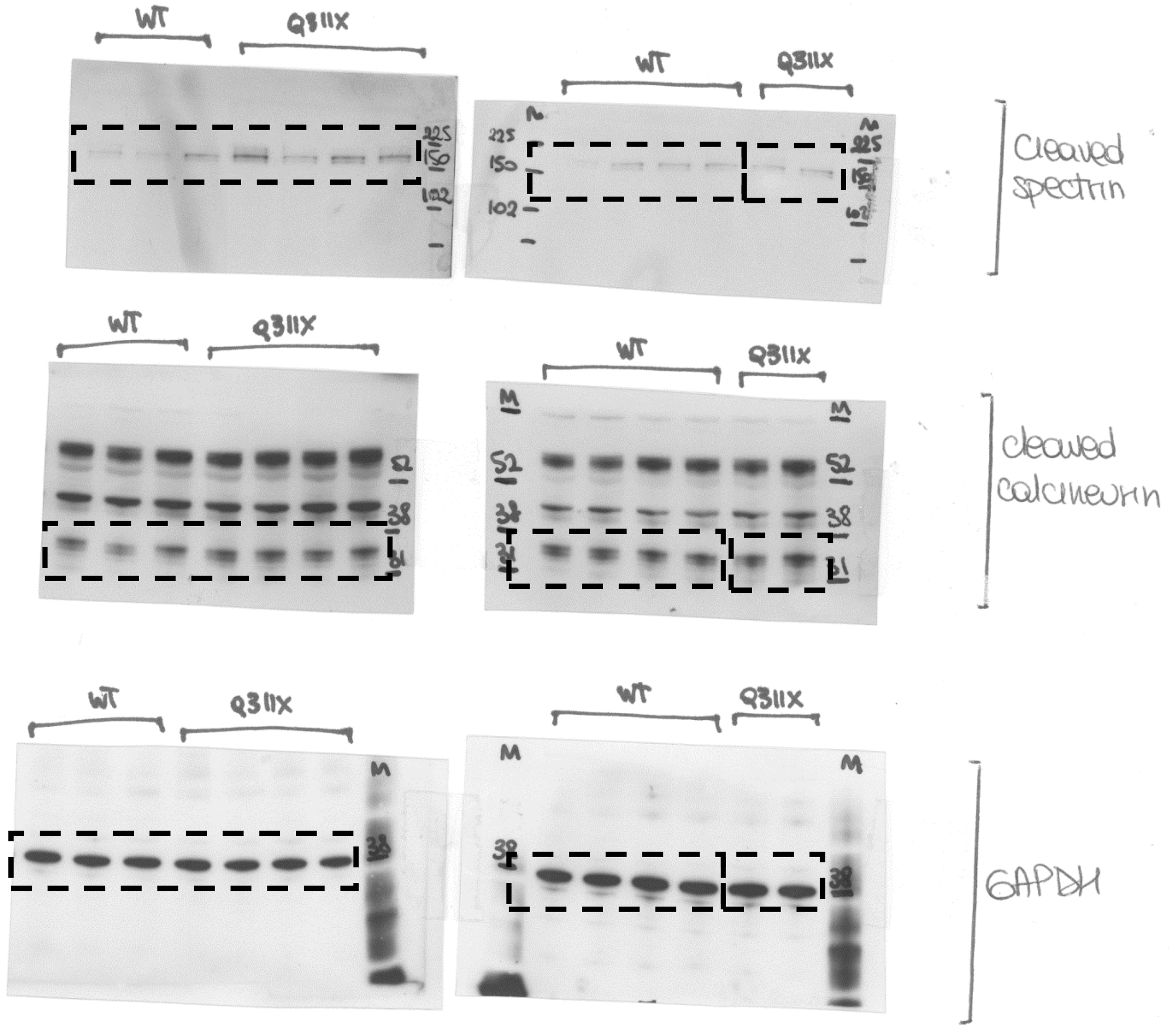

Supplementary Figure 12. Full blot images of western blots showed in Figure 4e.

Supplementary Table 1

Demographic and genetic data of the subjects included in the study.

SUBJECTS	AGE	SEX	DURATION	MUTATION
CONTROL 1	50	MALE		
CONTROL 2	65	MALE		
CONTROL 3	70	FEMALE		
CONTROL 4	45	FEMALE		
CONTROL 5	63	MALE		
ARJP PATIENT 1	63	MALE	30	PARK2 EXON 3 DELETION
ARJP PATIENT 2	68	MALE	35	PARK2 EXON 3 DELETION
ARJP PATIENT 3	62	MALE	38	PARK2 EXON 4 DELETION
ARJP PATIENT 4	71	FEMALE	49	PARK2 EXON 2/3 DELETION
